# Mechanical Properties of Bio-Composites Based on Epoxy Resin and Nanocellulose Fibres

**DOI:** 10.3390/ma14133576

**Published:** 2021-06-26

**Authors:** Martyna Roszowska-Jarosz, Joanna Masiewicz, Marcin Kostrzewa, Wojciech Kucharczyk, Wojciech Żurowski, Justyna Kucińska-Lipka, Paweł Przybyłek

**Affiliations:** 1 Faculty of Mechanical Engineering, Kazimierz Pulaski University of Technology and Humanities in Radom, E. Stasieckiego 54B Str., 26-600 Radom, Poland; m.jarosz@uthrad.pl (M.R.-J.); wojciech.kucharczyk@uthrad.pl (W.K.); wojciech.zurowski@uthrad.pl (W.Ż.); 2 Faculty of Chemical Engineering and Commodity Science, Kazimierz Pulaski University of Technology and Humanities in Radom, B. Chrobrego 27 Str., 26-600 Radom, Poland; m.kostrzewa@uthrad.pl; 3 Department of Polymer Technology, Faculty of Chemistry, Gdansk University of Technology, G. Narutowicza 11/12, 80-233 Gdansk, Poland; justyna.kucinska-lipka@pg.edu.pl; 4 Faculty of Aviation, Military University of Aviation, Dywizjonu 303/35 Str., 08-521 Dęblin, Poland; p.przybylek@law.mil.pl

**Keywords:** epoxy resins, nanocellulose preparation, mechanical properties, nanocomposites, sol-gel method

## Abstract

The aim of our research was to investigate the effect of a small nanocellulose (NC) addition on an improvement of the mechanical properties of epoxy composites. A procedure of chemical extraction from pressed lignin was used to obtain nanocellulose fibers. The presence of nanoparticles in the cellulose pulp was confirmed by FTIR/ATR spectra as well as measurement of nanocellulose particle size using a Zetasizer analyzer. Epoxy composites with NC contents from 0.5% to 1.5% *w*/*w* were prepared. The obtained composites were subjected to strength tests, such as impact strength (IS) and resistance to three-point bending with a determination of critical stress intensity factor (Kc). The impact strength of nanocellulose composites doubled in comparison to the unmodified epoxy resin (EP 0). Moreover, Kc was increased by approximately 50% and 70% for the 1.5 and 0.5% *w*/*w* NC, respectively. The maximum value of stress at break was achieved at 1% NC concentration in EP and it was 15% higher than that for unmodified epoxy resin. The highest value of destruction energy was characterized by the composition with 0.5% NC and corresponds to the increase of 102% in comparison with EP 0. Based on the analysis of the results it was noted that satisfactory improvement of the mechanical properties of the composite was achieved with a very small addition of nanofiller while other research indicates the need to add much more nanocellulose. It is also expected that this kind of use of raw materials will allow increasing the economic efficiency of the nanocomposite preparation process. Moreover, nanocomposites obtained in this way can be applied as elements of machines or as a modified epoxy matrix for sandwich composites, enabling production of the structure material with reduced weight but improved mechanical properties.

## 1. Introduction

Many scientists have discussed the influence of nanofillers on the properties of epoxy composites. [1,2] Cellulose contains micro and nanoscale fibers. The functional properties of nanocellulose fibers are determined by the significantly larger surface area in comparison to natural macrocellulose. This can cause stronger interactions with surrounding particles, e.g., it strongly binds its own and other nanoparticles. [3]

Over recent years, intensive research on innovative materials filled with nanocellulose has been carried out. This is due to the extraordinary properties of nanocellulose. Cellulose is a material widely occurring in nature which can be obtained from many natural sources, such as rice [4,5], wood, cotton and plant biomass [6]. Cellulose is also synthesized by algae and some bacteria. Other interesting sources of cellulose are the tunics of marine animals [7,8].

Biocomposites using epoxy resin as a matrix reinforced by cellulose combine the inherent properties of the reinforcement (biodegradability, renewability among others) with the properties of a high-performance resin. Composites containing nanocellulose fibers can be used in many applications. They can reduce the negative impact of polymer composites on the environment. The following are the scientific achievements to date in this field. Cellulose, which has been known for years, still surprises researchers with its applications, including automotive, packaging, electronics and sports industries, among others [9,10,11]. The development of science in this field allows for increasingly novel applications of nanocellulose in biomedicine [12,13]. Based on chemical modifications [14] and the analysis of the morphology of a nanocellulose [15], it is possible to obtain nanofibers that have significantly improved mechanical [16] and thermal properties, with reduced density and improved biocompatibility composites [17]. Considering these advantages, nanocomposites are also widely used in many industries [18]. The research on packaging containing nanocellulose is interesting [19]. It not only increases its mechanical strength but also reduces the content of phenols or anthocyanins, increases the antioxidant effects in fruit and protects against UV radiation [20]. Moreover, the advantages of nanocellulose allow it to be used as a reinforcement of structural composites, electronic component materials or water treatment materials [21]. The use of natural fillers has been described by many researchers as a very prospective development direction for biocomposites [22,23,24,25]. 

Wongjaiyen et al. [26] obtained nanofibres of cellulose (CNF) from sugar cane marc. They were then used for reinforcement in epoxy nanocomposites (0–10% of CNF weight). To obtain CNF, the cellulose was bleached with sodium chlorine, hydrothermal hydrolysis with oxalic acid at 800 psi at 100 °C in a microwave reactor was undertaken and it was homogenized mechanically. Tensile strength and Young’s modulus increased with the increase in CNF concentrations up to 3.0 weight % (increase by about 42% and 0.2 GPa). At higher CNF concentrations, modulus and tensile strength decreased. Flexural stress at break increased more while at the same time a decrease of Young’s modulus was observed. The obtained composite reached a maximum improvement of about 55% in comparison to EP 0.

Yeo and his collaborators [27] modified the surface of microfibrillated cellulose (MFC) with the use of triethoxy-(3-glicidylpropyl) silane (GPS). Epoxy composites reinforced with GPS-MFC and unmodified MFC were prepared. GPS-MFC/epoxy composites showed better mechanical properties than the initial MFC/epoxy ones. Mechanical properties of GPS-MFC/EP composites increased by up to 300% in comparison to pure epoxy resin. These were about 1.5–2.2-times higher than those of unmodified MFC/EP composites. The resistance to brittle cracking of the GPS-MFC/EP composite system has improved significantly. Critical stress intensity factor (Kc) and critical strain energy release rate (GIC) also increased by 1.5- and 5.8-times, respectively, in comparison to the reference MFC/EP composites. These results indicate an increased interfacial adhesion between cellulose filler and epoxy matrix.

Other scientists, Tang and Weder [28], prepared nanocomposites composed of cellulose nano-whiskers and epoxy resin. Cellulose with the shape factor of 10 and 84, respectively, was obtained from cotton and shields. The whiskers were introduced into dimethylformamide and added together to the epoxy resin. A curing agent based on diethyl toluenediamine was used. Thin films were cast and then cured. The content of nanocrystallites oscillated between 4 and 24 weight %. Electron microscopy confirmed that whiskers were evenly dispersed in the epoxy matrix. The tensile modulus for nanocomposites increased slightly, from 1.6 GPa for a pure polymer to 4.9 and 3.6 GPa for nanocomposites containing 16 wt. % of plant crystallites. The strength increased at 185 °C (i.e., above Tg), where the modulus increased from 16 MPa (pure polymer) to 1.6 GPa (casing) or 215 MPa (cotton). The mechanical properties of the new materials were the result of the formation of a percolating network of crystallites. The transfer of stresses was facilitated by strong interactions between them.

The main drawback of using plant fibers containing cellulose or nanocellulose in polymer composites is related to the hydrophilic character of the cellulose. Chemical surface modification is highly recommended to improve its compatibility with the hydrophobic polymer matrix. The resulting composite will thus be susceptible to moisture absorption that can bring dimensional changes and weaken interfacial adhesion, reducing the efficiency of stress transfer from the matrix to the reinforcement. Besides, due to its hydrophilic character, cellulose tends to form agglomerates during composite processing. Nanoparticles, particularly, have the natural tendency to agglomerate due to their high specific area and surface energy [29,30]. 

To reduce the referred incompatibility and extend its use to a range of highly sophisticated applications, the reinforcement surface can be chemically modified. Chemical treatments can increase interfacial adhesion with epoxy and decrease water absorption. Indeed, cellulose has three hydroxyl groups per repeating unit, which makes it readily susceptible to chemical modifications. Among the reported chemical methods, mercerization, silanization, oxidation, esterification, grafting of branched/hyperbranched polymers stand out. [29,30,31,32,33,34,35] In our work we modified the method to obtain nanocellulose fibers composed of cellulose acetate. The developed preparation method enabled the direct addition of NC dispersion to EP, whereby we have eliminated the problem of incompatibility of raw materials at the production stage.

Based on the literature, most researchers report that for epoxy nanocomposite improvement it is recommended to add a larger amount of micro and nanocellulose. [36,37,38,39] The purpose of our research was to investigate the effect of a small nanocellulose addition on the mechanical properties of epoxy composites. In this case, it was prepared extract nanocellulose particles from compressed lignin. A major advantage of our work is that we obtained nanocellulose as concentrated dispersion in acetone which can be easily and directly used for nanocomposite preparation. There were no observed problems with mixing/homogenization of the hydrophobic resin with hydrophilic nanocellulose. The composites were prepared with different nanofiller content (from 0.5% to 1.5% by weight) to determine the lowest optimal amount of nanocellulose which can improve the mechanical properties of pristine epoxy resin. Moreover, obtained epoxy nanocomposites contained the effective nanofiber reinforcement prepared from wooden biomass.

## 2. Materials

All materials that were used to prepare nanocellulose and samples of epoxy nanocomposites are listed in Table 1.

### 2.1. Preparation of Nanocelulosse

An obtainment procedure of cellulose acetate nanoparticles based on the literature [40] was modified. The developed preparation method enabled direct addition of NC dispersion to EP as well as easy and excellent incorporation into EP. First, 50 g of lignin was shredded into small pieces. Then, the pieces were pre-wetted in a 2% NaOH solution. In the next stage, cellulose lignin was drained from the solution and washed with distilled water. Then, it was mercerized using 10% NaOH solution at boiling point for 1.5 h. This was repeated 3 times. After each step, the cellulose was filtered and washed with distilled water until a constant pH value was achieved.

After mercerization, the cellulose bleaching process was started. The cellulose was placed in distilled water and heated with the addition of a 7 portion of acetic acid and solution sodium chlorate (V). Next, the obtained pulp was filtered and washed with distilled water until a constant pH was obtained. Afterward, the cellulose pulp was placed in a flask together with a mixture of acetic acid and nitric acid (V). The mixture was brought to boiling point and heated for 1.5 h.

After this step, the mixture was cooled by dilution in cold distilled water. The content of a flask was decanted and filtered. The accumulated pulp was transferred to centrifuge tubes. To get rid of the remaining acid solution, distilled water was added to each test tube. Then, the contents were mixed and centrifuged; the procedure was repeated twice. In the last stage, instead of water, acetone was added to the cellulose pulp. The mixture was centrifuged three times to obtain a smooth and cream-like cellulose dispersion in acetone. In Table 2 there is a list and the amounts of raw materials used to obtain cellulose acetate nanofibers.

### 2.2. Preparation of Reference Samples

Epoxy resin in the amount of 89.3 g was introduced into a 250 cm^3^ beaker. Z1 hardener was added in stoichiometric amount. The whole mixture was mixed slowly for 5 min to avoid aeration. All compositions were placed into 10-sided steel molds coated with a release agent. Samples were allowed to cure and vent for 24 h at room temperature. After this time, the post-curing process was carried out at 80 °C for 3 h.

### 2.3. Preparation of Nanocelulosse EP + NC Samples

A weighted amount of epoxy resin was placed in marked beakers of 250 cm^3^ capacity. Then, an appropriate amount of nanocellulose suspension was introduced into individual beakers with resin. The system was heated up to 55 °C and thoroughly mixed and homogenized using mechanical stirrer IKA T18 (Staufen, Germany). Homogenization time: 10 min. In the next step the acetone was removed by evaporation (rapid stirring in a water bath, temperature: 90 °C, time: 1 h). Ultrasonic homogenization was applied using Hielscher UP 200H (Teltow, Germany). Homogenization time: 10 min, amplitude 100%, cycle-1. After cooling to 20 °C, a stoichiometric amount of hardener was added. The composition was mixed for about 5–10 min and placed in a 10-cavity mold, previously covered with an anti-adhesive agent. The samples were placed for curing at room temperature for 24 h. Then, they were post-cured at 80 °C for 3 h. Table 3 lists the contents of the various components used to prepare the composites.

## 3. Methods

### 3.1. Spectral Testing Methods

#### 3.1.1. Particle Size Measurements of Nanocellulose

The Zetasiner Nano 9 (Worcestershire, UK) was used to investigate particle size measurements of nanocellulose. A total of 1.0 g of cellulose was taken from the cellulose pulp. The sample was placed in a 50 cm^3^ beaker and diluted with distilled water. The mixture was homogenized in an ultrasonic stirrer for 30 min. After that time, the mixture was cooled down to 25 °C. The appropriate amount was transferred with a syringe to a polystyrene measuring cell. The test procedure was repeated three times.

#### 3.1.2. FTIR ATR

The analysis of the dry pulp of nanocellulose was performed using a FTIR Tensor 27 Bruker Co. spectrometer (Leipzig, Germany), in the range of 4000–400 cm^−1^. The samples were scanned at the resolution of 4 cm^−1^. FTIR-ATR analysis confirmed the chemical structure of nanocellulose.

#### 3.1.3. SEM

The morphology changes between unmodified and modified polymers were studied by FEI QUANTA 250 FEG SEM microscope (Thermo Fisher Scientific, Waltham, MA, USA) at an accelerating voltage equal to 10 kV and magnification of ×500 and ×5000. The pictures were taken from the surface area near the crack tip for the samples after the impact test.

### 3.2. Mechanical Properties Testing Methods

#### 3.2.1. Impact Strength

The impact strength of the specimens for individual compositions was tested with the Zwick 5012 (Ulm, Germany), according to ISO 179 [41]. The sample bars (80 mm × 10 mm × 4 mm), with notch 1 mm and supported with the span distance of 60 mm, were hit with a 2 J energy hammer. Three samples of each composite were used for the test. The results are presented in Section 4.2.

#### 3.2.2. Resistance to Crack Propagation

Measurement was carried out in a three-point bending mode for rectangular (80 mm × 10 mm × 4 mm) noched (1 mm) samples using the Zwick/Roell apparatus (Ulm, Germany). The support spacing of 60 mm and a bending speed of 5 mm/min at a room temperature were applied. The critical stress intensity factor (Kc) was calculated using the formulas [42,43]:

(1) $$K_{C}=\frac{3\;\cdot P\;\cdot L\;\cdot \;\sqrt{a}}{2\;\cdot t\;\cdot \;w^{2}}\;\cdot Y\left(\frac{a}{w}\right)\left[\mathrm{MPa}\cdot \sqrt{m}\right],$$

where *P* is the load at fracture [N], *L* is the distance between the spans [m], *a* is the notch length [m], *t* is the sample’s thickness [m], *w* is the sample’s width [m] and *Y* is the geometry-dependent factor.

(2) $$Y\left(\frac{a}{w}\right)=1.93-3.07\left(\frac{a}{w}\right)+14.53{\left(\frac{a}{w}\right)}^{2}-25.11{\left(\frac{a}{w}\right)}^{3}+25.80{\left(\frac{a}{w}\right)}^{4}$$

#### 3.2.3. Resistance to Three-Point Bending

Measurement was performed using the Zwick/Roell apparatus. Three samples from each composition were subjected to three-point bending according to ISO 178 [44]. The beams were supported on 60 mm spacing supports at a bending speed of 5 mm/min.

### 3.3. Thermal Testing Methods

#### 3.3.1. TGA

The TGA test for unmodified and modified polymers was made using the Netzsch TG 209F3 apparatus (Thermogravimetric Analyzer, NETZSCH-Gerätebau GmbH, Selb, Germany). Samples were tested in the temperature range from 30–800 °C at a rate of 10 °C/min. The measurement was carried out in a nitrogen atmosphere. For obtained epoxy nanocomposites containing different NC loading, characteristic temperatures for 2, 5, 10 and 50% of weight loss were determined.

#### 3.3.2. DSC

For all samples a DSC test was made on the Netzsch DSC 204F1 Phoenix apparatus (Differential Scanning Calorimeter, NETZSCH-Gerätebau GmbH, Selb, Germany). DSC analysis was performed in the temperature range from 20–200 °C at a rate of 10 °C/min. The measurement was performed in an inert nitrogen atmosphere. Samples of about 5–10 mg were crimp-sealed in aluminum pans. Two heating runs were carried out and cooling was performed spontaneously.

Based on the above-described test methodology, the samples of all compositions were tested. The results in the form of averaged values are presented in the form of graphs or tables.

## 4. Results

### 4.1. Spectral Testing Results

**Figure 1:**
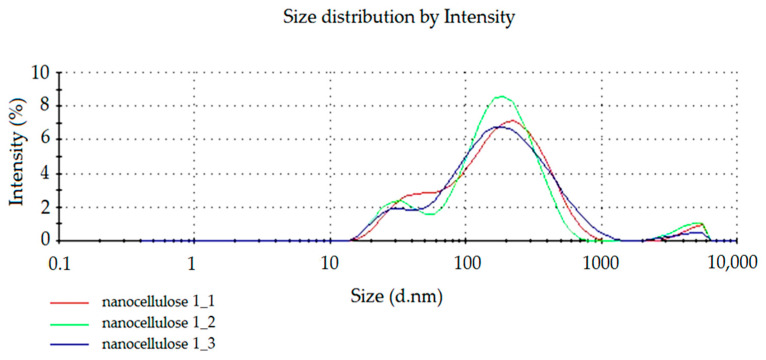
The spectrum of dependence of particle intensity on particle size.

**Figure 2:**
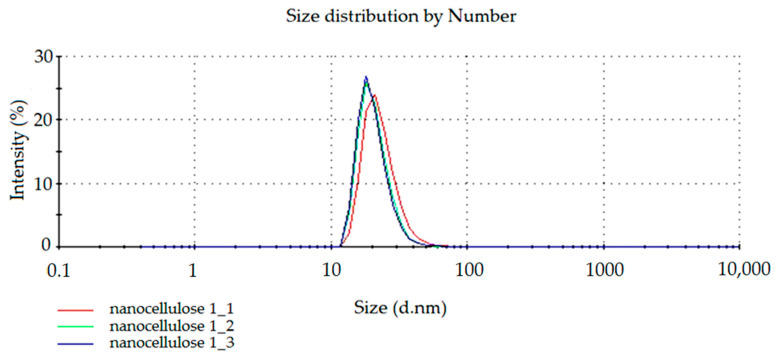
The spectrum of dependence of the number of particles on their size.

To confirm the presence of cellulose nanoparticles within the prepared dispersion, the particle size was measured using the Zetasizer Nano 9 (Worcestershire, UK). The spectra of the light reflection intensity and the number of nanocellulose particles in water dispersion are presented below. In Figure 1 and Figure 2, the results of measurements repeated in triplicate (nanocellulose 1_1, nanocellose 1_2, nanocellose 1_3) for the obtained NC dispersion are shown.

From the diagram (Figure 1) of the dependence of the intensity of particles on their size, it can be shown that the highest intensity is in the 240.6 nm range. Particles in the size range of 30.89 nm show an intensity of about 11%. A small peak size of 4 μm can also be observed.

From the particle size dependence diagram (Figure 2) it was shown that in the samples used for testing, there are particles in the 20 nm range in prevalence to others.

**Figure 3:**
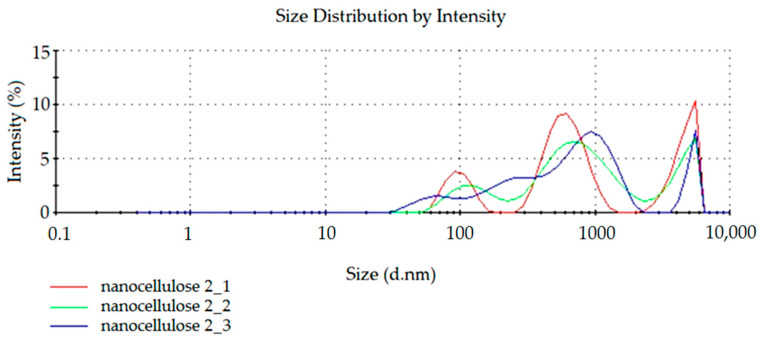
The spectrum of dependence of particle intensity on particle size.

**Figure 4:**
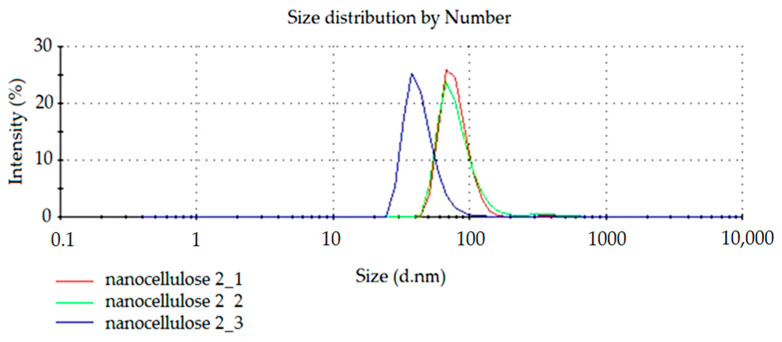
The spectrum of dependence of the number of particles on their size.

To confirm the repeatability of the modified NC preparation method, the process was carried out for the second time. The results of light reflection intensity spectra for NC dispersion obtained in the repeated process are presented in Figure 3 and Figure 4. The tests were also carried out three times (curves: nanocellulose 2_1, nanocellulose 2_2 and nanocellulose 2_3).

In this case (Figure 3), an increased intensity of peaks within 700 nm and 5 μm was observed. There are also peaks of particles below 100 nm, although with a reduced intensity compared to larger particles. This phenomenon can be explained by the formation of agglomerates of cellulose nanoparticles.

The diagram of the dependence of the number of particles on their size (Figure 4) analysis shows that most of the particles have a size below 100 nm.

Based on the analysis of results from both carried out NC preparation processes we can conclude that our process enables us to obtain a large fraction of cellulose in nanometric form.

**Figure 5:**
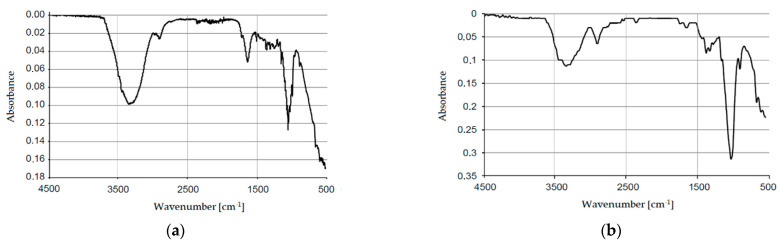
Results of FTIR/ATR tests: (**a**) FTIR/ATR spectrum (wet nanocellulose); (**b**) FTIR/ATR spectrum (dry nanocellulose).

The obtained nanocellulose was also examined by infrared spectroscopy. FTIR/ATR tests were carried out to determine the chemical structure of the obtained product (nanocellulose). The resulting spectra are illustrated in Figure 5a,b.

The analysis of the obtained infrared spectra shows in both cases bands with wavelengths of 3330 cm^−1^ and 2900 cm^−1^, characteristic for O-H and C-H groups. The peak within 1735 cm^−1^ from ester groups is also important. The peak within 1640 cm^−1^, corresponding to the vibration of hydroxyl groups from absorbed water, was also observed on the wet nanocellulose spectrum. Peaks typical for C-H and O-H groups, from the polysaccharide ring, were observed for both spectra in the range of 1370 cm^−1^. A 1060 cm^−1^ wavelength band is characteristic for C-O-C groups and occurs in the polysaccharide ring. The interpretation of the obtained spectra shows the formation of acetylated cellulose.

### 4.2. Mechanical Testing Results

A series of mechanical tests were performed with the prepared composite samples containing nanocellulose as a reinforcement for the epoxy matrix. The resistance for crack propagation was determined based on the impact strength and critical stress intensity factor tests. The results are shown in Table 4

The results obtained in Table 4 show clearly that the addition of nanocellulose improves impact strength and Kc of epoxy composites. A double increase in impact strength was achieved with Ep0 + 1.5% NC. The other samples improved their impact strength by about 70–90% compared to the reference composite. Unmodified epoxy resin is a brittle material. The addition of nanocellulose caused an increase in impact strength, indicating an increase in the material resistance to cracking under dynamic load. For all modified composites an increase in the critical stress intensity factor was observed. The highest value was reached by the composition with 0.5% NC, which is an improvement of resistance to brittle cracking by 70%.

For explanation of the observed impact strength improvement, SEM micrographs were taken from the impact fractured surfaces, near the crack tip, for pristine epoxy resin samples and compositions containing nanocellulose.

**Figure 6:**
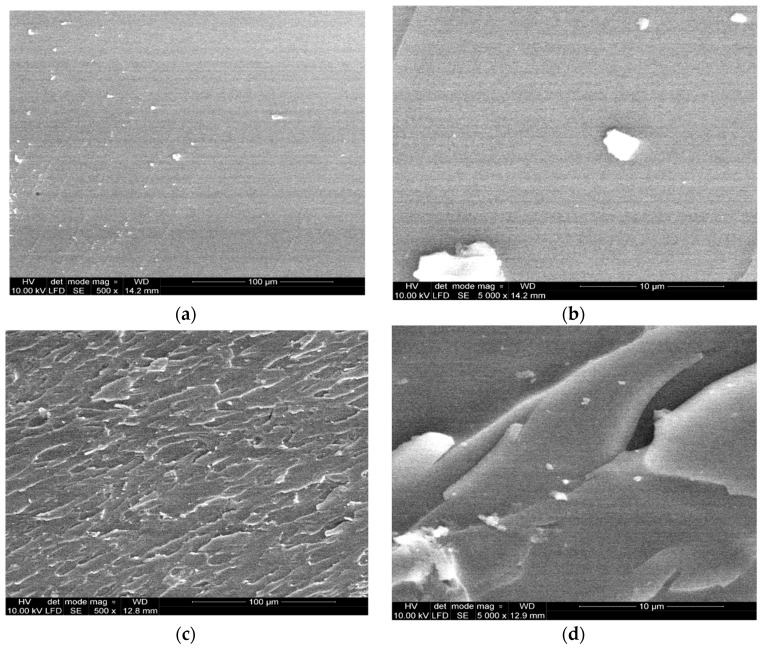
SEM micrograph of the fracture surface of EP0 (**a**,**b**) and EP modified with 1% NC (**c**,**d**).

The micrograph of the unmodified epoxy composition fracture surface is flat and glassy (Figure 6a,b), indicating the occurrence of a regular uninterrupted, glassy surface of a crack propagation path. It looks typical for the brittle behavior of materials. It is usually used to explain the low fracture energy values as well as low impact strength of unmodified epoxy systems [45]. In comparison to the pristine epoxy resin surface micrographs, epoxy nanocomposites exhibit considerably different morphologies and microstructures. The fracture surface of EP containing 1 wt% nanocellulose is rough with the presence of uniform leaflike plastic yielding and cracking areas within the polymer matrix (Figure 6c,d). The SEM pictures of the 1% NC composition show some individual fractured nanofiber ends as white dots and microcrack edges. The extremely short pull-out lengths indicate strong interfacial adhesion and strong molecular interactions.

The cellulose nanofibers appear as quite well dispersed individual entities in the epoxy matrix. We can also observe some larger particles among the crack surface area. This can be attributed to some aggregate particles on NC occurrence detected and described in the Zetasizer results analysis. It is well accepted that the occurrence of plastic shear yielding within a polymer matrix explains the increase of its mechanical and adhesive properties [36,46].

**Figure 7:**
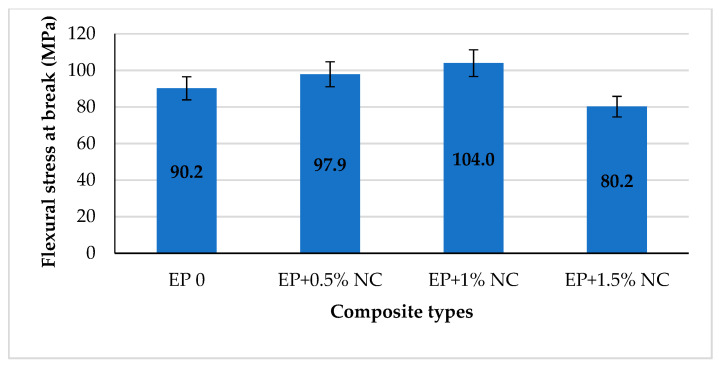
Flexural stress at break for epoxy composites with NC.

In the next step, the graph of the dependence of flexural stress at break of the composition was analyzed and is shown in Figure 7.

Flexural stress at break is a parameter that determines the value of the force needed to break the sample. The graph shows that it increases to 1% NC content and then decreases as the number of modifiers increases. The maximum flexural stress at break was obtained at 1% NC content, about 15% higher than unmodified epoxy resin. Therefore, the strength properties of the sample increase to a certain limit amount of nanofiller. Too much nanocellulose has a negative effect on the sample properties.

**Figure 8:**
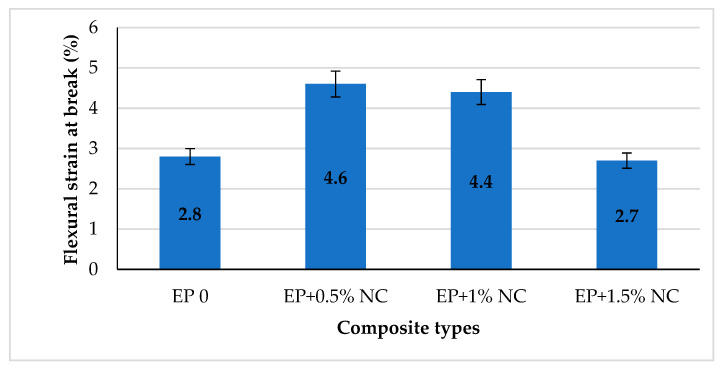
Flexural strain at break for epoxy composites with NC.

Figure 8 summarizes the results of the dependence of flexural strain at break with different nanocellulose contents.

**Figure 9:**
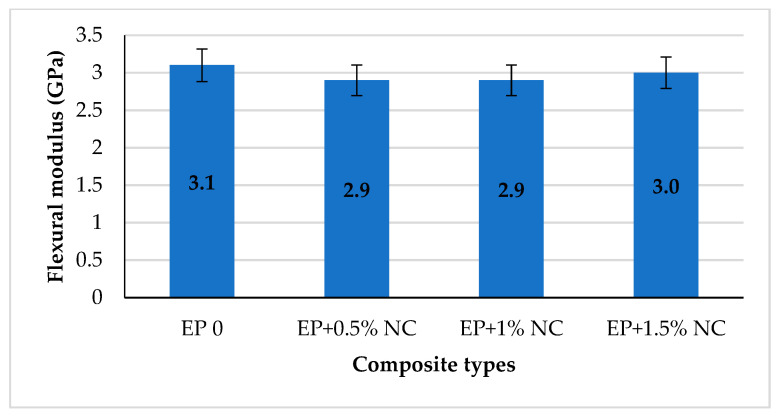
Flexural modulus for epoxy composites with NC.

Based on the values shown in the graph it was shown that a small addition of cellulose nanofiller contributes to an increase of the flexural strain at break. A maximum increase of about 64% was observed for 0.5% NC in comparison with EP 0. Then, a slight decrease in strain value was observed for composite with 1% on NC and rapid drop-down for EP + 1.5% NC. This might be attributed to the larger amount of well-dispersed cellulose nanoparticles within the epoxy matrix, as well as the formation of strong interaction between EP and NC [29,47]. This is also confirmed by the flexural modulus values shown in Figure 9.

**Figure 10:**
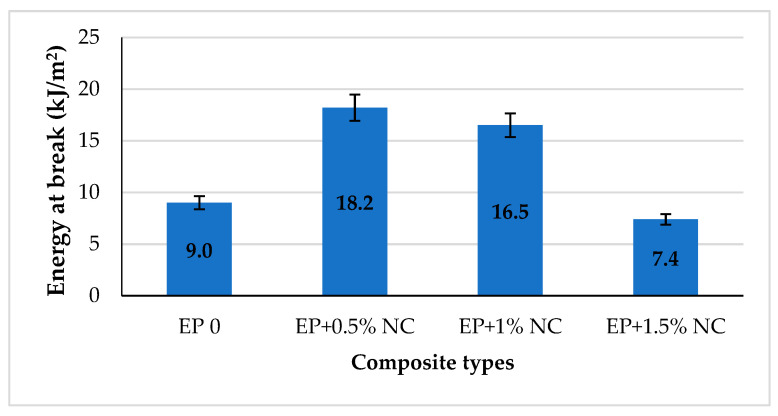
The energy at break for epoxy composites with NC.

All tested compositions containing nanocellulose were characterized by lower modulus of bending in comparison with unmodified resin. The decrease in value is estimated at 10%. This indicates that modified compositions have become a little more flexible than EP 0. In conclusion, nanocellulose does not change the stiffness of the obtained epoxy composite but significantly improves the resistance to three-point bending and strength of epoxy resin with 0.5–1% *w*/*w* NC loading. This can be also proved by the comparison of the energy at break values in Figure 10.

According to the results presented in Figure 10, the highest value of energy at break was in the composition with 0.5% NC and corresponds to an increase of 102% in comparison with EP 0. As we can see, incorporation of 0.5% and 1% *w*/*w* of nanocellulose within epoxy resin can significantly enhance the strength of the obtained nanocomposite, because more energy is needed to break the sample. Only for composite EP + 1.5% NC was no improvement in the energy at break value detected in comparison with unmodified epoxy resin. Even the value of energy at break is lower, at about 20% of that for neat epoxy resin. Thus, it is important not to overload the matrix with nanofiller, which can strongly interact with the matrix. Moreover, it is well known that the incorporation of solid particles in the matrix causes an increase in the material brittleness [48,49]. In the case of nanoparticles, especially in high concentration, we can also observe the strong tendency to aggregate formation, which can act as stress concentration points in the material [30]. 

### 4.3. Thermal Testing Results

**Figure 11:**
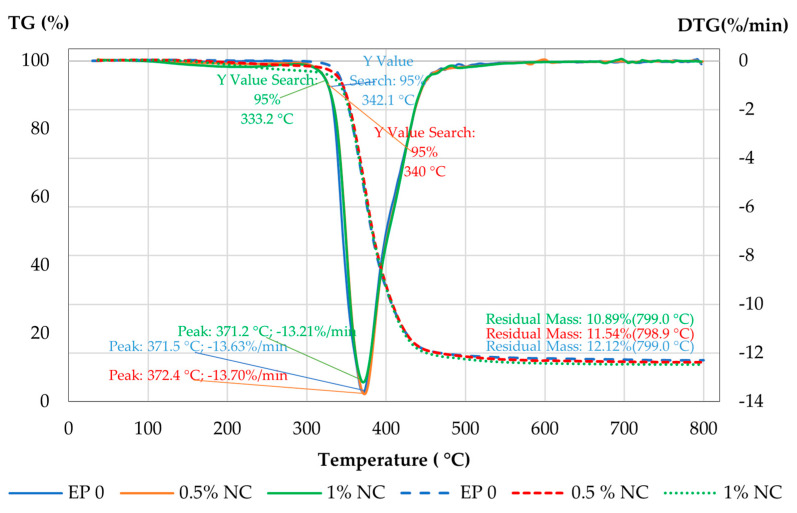
Comparison of TGA for NC composites.

The thermal properties of the obtained biocomposites were determined by thermogravimetric analysis. The thermal stability and resistance of NC composites are very important for many applications. It is necessary to characterize and determine the maximal temperature which can be applied to the new composite material. The effect of different contents of NC on thermal degradation was investigated. The TGA curves for all composites are shown in Figure 11.

The observed sudden drop in the TGA curves in the mass of the sample indicated the thermal degradation of the material. The materials started to thermally degrade at 330 °C and decomposed at 440 °C. At this temperature, a substantial loss in their weights was observed. The heat initiated the degradation processes and the breaking down of the fibers and matrix structure. This caused a molecular chain rupture or scission.

Based on the analysis of the weight loss rate (DTG, %/min), detailed data on the thermal degradation of the composites with NC were collected and are presented in Table 5. These data show that the introduction of NC fibers into the epoxy matrix did not significantly alter the degradation temperature of the composites.

**Figure 12:**
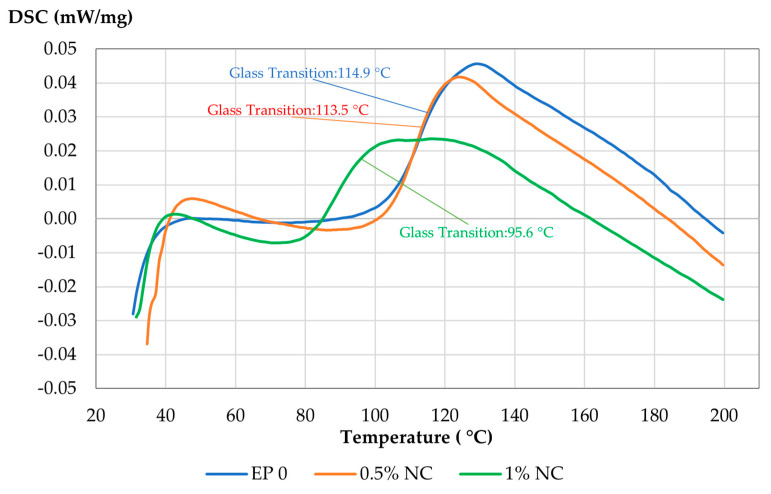
DSC thermogravimetric curves for composites with nanocellulose.

The DSC analysis was performed in a heating–cooling–heating cycle. Figure 12 presents the results of the second heating. During the first heating, the samples were heated to 200 °C, then left to cool down to reach room temperature and heated up again to 200 °C with a heating rate of 10 °C/min.

DSC analysis allows determining the effect of nanofiller on the glass transition temperature (Tg) of the obtained NC composites. Based on the analysis of the results presented above it can be observed that there are some shifts to lower Tg values for composites containing NC. On DSC curves (the second heating cycle), glass transition for EP0 equals 115 °C, 113.5 °C for composite with 0.5% *w*/*w* of NC and 95.6 °C for composite containing 1% of nanocelluose fibers. It can be noted that with the nanocellulose concentration increase, the glass transition temperature decreased. The decrease in Tg may be partially attributed to incomplete evaporation of the solvent during the composite preparation. It was also observed that NC has a little plasticizing effect on epoxy resin. This is in accordance with the flexural properties results and can explain the observed increase in strain at break) for composites containing 0.5–1.0% of NC.

## 5. Discussion

The analysis of the obtained results confirms that the addition of a small (0.5–1.0% *w*/*w*) amount of nanocellulose can significantly improve the mechanical properties of epoxy resin. Verification of the test results showed that all nanocellulose-modified composites had better critical stress intensity and the presence of nanocellulose at 0.5% improved this parameter by about 70%. The above-mentioned data also confirm that the material’s resistance to three-point bending is higher considering the reference composite. This was evidenced by the higher energy to break and stress at the break values (102% and 15%, respectively) in comparison to EP0. The obtained composites can be used as new construction materials containing an effective nanofiller made of cheap and easily available plant material. They can be used for machine elements or covers production and can be applied as a modified epoxy matrix for sandwich composites reinforced with glass or aramid textile.

Significant improvement of the composite resistance in comparison to unmodified epoxy resin can be attributed to well-dispersed NC and possible good interaction as well as a chemical reaction between epoxy resin functional group and hydroxyl or acetyl groups from nanocellulose [29]. Suggesting a few tens of percent improvement in strength parameters with a tiny filler addition, we can conclude that it does not act as an ordinary filler, but as a nanofiller. A major advantage of our work is the obtained nanocellulose concentrated dispersion in acetone which can be directly and easily used for nanocomposite preparation. In this case we have not observed problems with the mixing or homogenization of hydrophobic resin with hydrophilic nanocellulose. It is important to use as little acetone as possible in the dispersion preparation and to remove it, as thoroughly as possible, before adding it to the resin. The obtained results are very promising in composite manufacturing. Despite many scientific reports which used larger amounts or mixtures of solvents [24,26], associated with large raw material losses and time-consuming operations, we prepared epoxy nanocomposite with significantly improved strength properties. This was achieved by a small amount of natural nanofiller (0.5–1 wt%) and less solvent.

## 6. Conclusions

Based on the obtained results we can conclude that we can prepare NC by acid hydrolysis combined with delignification with good yield. Moreover, during the preparation process we obtained concentrated NC dispersion in acetone which can be directly used for epoxy resin modification. The obtained nanocomposites with 0.5–1% *w*/*w* NC loading exhibited significantly improved mechanical properties in comparison to unmodified epoxy resin expressed by:

- impact strength higher by about 70–90%;
- an improvement of resistance to brittle cracking by 70%;
- flexural stress at break higher about 15%;
- higher energy to break and stress at the break i.e., for the best compositions 102% and 15%, respectively.

The significant improvement of properties with a very small amount of additive might be attributed to the presence of well-dispersed nanoparticles of NC in the epoxy resin matrix and good chemical compatibility between epoxy resin functional groups and NC. The fracture surfaces of epoxy composites, as presented by SEM micrographs, showed delaminated and stratified structures which can explain the significant increase in the impact strength and toughness of tested composites containing NC as a nanofiller. Thermal tests show relatively good resistance of the materials at elevated temperatures.

## Figures and Tables

**Table 1:** Materials used for testing.

Material	Description
Epoxy resin	Low-molecular-weight epoxy resin Epidian 5, Bisphenol A diglycidyl ether, Viscosity at 25 °C: 20,000–30,000 mPa∙s Epoxide equivalent weight: 196–208 g/mol Epoxide number: 0.480–0.510 mol/100 g Density at 20 °C: 1.17 g/cm^3^
Hardener	Z1 (triethylenetetramine)
Lignin	Thinly pressed cellulose sheets
Sodium hydroxide	Molecular weight: 40 g/mol Characteristics: white granules
99.5% acetic acid	Molecular weight: 60 g/mol Characteristics: High concentration solution
65% nitric acid (V)	Molecular weight: 63 g/mol Characteristics: Colorless solution
Sodium chlorate (V)	Molecular weight: 106 g/mol Characteristics: white powder
Acetone	Molecular weight: 58 g/mol Characteristics: liquid

**Table 2:** Reagents used for NC preparation.

Reagent	Volume (mL)	Number of Moles
Sodium hydroxide (2% water solution)	1500	0.75
Sodium hydroxide (10% water solution)	4500	11.25
Acetic acid, pure 95.5%	956	16.65
Nitric acid (V), pure 65%	150	2.17
Sodium chlorate (V) (25% water solution)	280	0.79
Acetone, pure 99.5%	238.6	2.03

**Table 3:** Content of the compositions.

Composition	Epoxy Resin Epidian 5 (g)	Hardener (g)	Nanocellulose (g)
Ep 0	89.3	10.7	0
Ep + 0.5% NC	88.8	10.7	0.5
Ep + 1% NC	88.4	10.6	1
Ep + 1.5% NC	88.0	10.5	1.5

**Table 4:** Results of impact tests and values of critical stress intensity factor calculation.

Composites	Impact Strength (kJ/m^2^)	Kc (MPa∙m^1/2^)
Ep 0	0.9	1.5
Ep + 0.5% NC	1.7	2.6
Ep +1% NC	1.5	2.5
Ep + 1.5% NC	1.9	2.3

**Table 5:** Weight loss in NC composites at different temperatures.

Weight Loss [%]	Degradation Temperature [°C]		
	EP 0	EP + 0.5% NC	EP + 1% NC
2	333.2	317.8	235.3
5	342.1	340.0	333.2
10	349.0	349.7	347.5
50	381.7	382.7	382.2

## Data Availability

Not applicable.
